# Plasma neurofilament light, glial fibrillary acid protein, and phosphorylated tau 181 as biomarkers for neuropsychiatric symptoms and related clinical disease progression

**DOI:** 10.21203/rs.3.rs-4116836/v1

**Published:** 2024-03-22

**Authors:** Miriam Rabl, Leonardo Zullo, Piotr Lewczuk, Johannes Kornhuber, Thomas K Karikari, Kaj Blennow, Henrik Zetterberg, Francesco Bavato, Boris B Quednow, Erich Seifritz, Armin von Gunten, Christopher Clark, Julius Popp

**Affiliations:** Department of Adult Psychiatry and Psychotherapy, Psychiatric University Hospital Zurich, University of Zurich; Department of Psychiatry, Old Age Psychiatry Service, Lausanne University Hospital; Department of Psychiatry and Psychotherapy, Universitätsklinikum Erlangen and Friedrich-Alexander Universität Erlangen-Nürnberg; Department of Psychiatry and Psychotherapy, Universitätsklinikum Erlangen and Friedrich-Alexander Universität Erlangen-Nürnberg; Department of Psychiatry and Neurochemistry, Institute of Neuroscience & Physiology, the Sahlgrenska Academy at the University of Gothenburg; Department of Psychiatry and Neurochemistry, Institute of Neuroscience & Physiology, the Sahlgrenska Academy at the University of Gothenburg; Department of Psychiatry and Neurochemistry, Institute of Neuroscience & Physiology, the Sahlgrenska Academy at the University of Gothenburg; Department of Adult Psychiatry and Psychotherapy, Psychiatric University Hospital Zurich, University of Zurich; Experimental and Clinical Pharmacopsychology, Department of Adult Psychiatry and Psychotherapy, Psychiatric University Hospital Zurich, University of Zurich; Department of Adult Psychiatry and Psychotherapy, Psychiatric University Hospital Zurich, University of Zurich; Department of Psychiatry, Old Age Psychiatry Service, Lausanne University Hospital; Department of Adult Psychiatry and Psychotherapy, Psychiatric University Hospital Zurich, University of Zurich; Department of Adult Psychiatry and Psychotherapy, Psychiatric University Hospital Zurich, University of Zurich

**Keywords:** Neuropsychiatric symptoms, blood-based biomarkers, Alzheimer’s disease, cognition, phosphorylated tau 181, pTau181, neurofilament light chain, NfL, glial fibrillary acid protein, GFAP

## Abstract

**BACKGROUND:**

Neuropsychiatric symptoms (NPS) are common in older people, may occur early in the development of dementia disorders, and have been associated with faster cognitive decline. Here, our objectives were to investigate whether plasma levels of neurofilament light chain (NfL), glial fibrillary acid protein (GFAP), and tau phosphorylated at threonine 181 (pTau181) are associated with current NPS and predict future NPS in non-demented older people. Furthermore, we tested whether the presence of NPS combined with plasma biomarkers are useful to predict Alzheimer’s disease (AD) pathology and cognitive decline.

**METHODS:**

One hundred and fifty-one participants with normal cognition (n=76) or mild cognitive impairment (n=75) were examined in a longitudinal brain aging study at the Memory Centers, University Hospital of Lausanne, Switzerland. Plasma levels of NfL, GFAP, and pTau181 along with CSF biomarkers of AD pathology were measured at baseline. NPS were assessed through the Neuropsychiatric Inventory Questionnaire (NPI-Q), along with the cognitive and functional performance at baseline and follow-up (mean: 20 months). Linear regression and ROC analyses were used to address the associations of interest.

**RESULTS:**

Higher GFAP levels were associated with NPS at baseline (β=0.23, p=.008). Higher NfL and GFAP levels were associated with the presence of NPS at follow-up (β=0.29, p=.007 and β=0.28, p=.007, respectively) and with an increase in the NPI-Q severity score over time (β=0.23, p=.035 and β=0.27, p=.011, respectively). Adding NPS and the plasma biomarkers to a reference model improved the prediction of future NPS (AUC 0.73 to 0.84, p=.007) and AD pathology (AUC 0.79 to 0.86, p=.006), but not of cognitive decline (AUC 0.79 to 0.84, p=.068).

**CONCLUSION:**

Plasma GFAP is associated with NPS while NfL and GFAP are both associated with future NPS and NPS severity. Considering the presence of NPS along with blood-based AD-biomarkers may improve diagnosis and prediction of clinical progression of NPS and inform clinical decision-making in non-demented older people.

## INTRODUCTION

Neuropsychiatric symptoms (NPS) are common in older people, with rates of 80% or more in patients with cognitive impairment and dementia (1). NPS frequently occur already at preclinical or prodromal stages of Alzheimer’s disease (AD) (2, 3) and therefore can be seen as a risk factor for the progression to dementia (4–6). NPS have been associated with lower quality of life, more frequent hospitalizations, and earlier death (7–9). Early detection and evaluation of NPS is therefore important for a more accurate prognosis and more specific treatment interventions to lower disease burden and potentially improve long-term outcomes.

NPS are generally assessed by clinical examination and by interviewing the patients or their caregivers about single symptoms like apathy, depression, irritability, or agitation (10). There is an important inter-individual heterogeneity regarding the causes and factors contributing to the manifestation and duration of NPS, and it is generally difficult to estimate whether the observed symptoms are mainly related to acute factors like psychological stress, and/or pathophysiological changes. Easily available biomarkers indicating NPS-related pathology and the associated risk for long-term consequences could enable a more accurate etiological diagnosis and prognosis in patients having NPS.

To date, little is known about the underlying pathophysiology of NPS. Some studies investigated associations of NPS with AD using cerebrospinal fluid (CSF) biomarkers or brain PET-imaging. One study found that apathy correlated with increased levels of phosphorylated and total tau in CSF (11), another one reported increased aggressive behavior in AD patients in association with lower beta-amyloid 1–42 peptide (Aβ_42_) levels (12). However, a meta-analysis of 21 studies addressing the association of NPS with CSF biomarkers of AD pathology showed inconsistent findings (13). A study using tau- and amyloid PET analysis found that NPS correlate with tau but not with amyloid pathology (14). In a study in the Alzheimer’s Disease Neuroimaging Initiative (ADNI) cohort some symptoms such as apathy, anxiety, and delusions were associated with amyloid pathology in participants with mild cognitive impairment (MCI), but not in cognitively unimpaired subjects or patients with dementia (15).

PET imaging and CSF analysis are based on expensive and/or invasive procedures. Thus, there is a need for biomarkers easier to obtain such as blood-based markers, to detect pathology and to predict or track disease progression in clinical settings. Neurofilament light chain (NfL) has been proposed as a biomarker for axonal damage and neuronal injury of different etiologies, including AD (16). Glial fibrillary acid protein (GFAP) is a marker of astrocytosis and astroglial activation and has also been related to AD and early stages of AD (17, 18). Another blood-based biomarker candidate is tau phosphorylated at threonine 181 (pTau181), which indicates cerebral tau pathology specific for AD (19). All three blood markers have been shown to be useful for detecting and monitoring neurodegeneration (20–24). Some studies also evaluated NfL and GFAP in the blood as potential markers for primarily psychiatric disorders such as depression or schizophrenia (25–27), but little or inconsistent evidence of the associations between those plasma biomarkers with NPS in the context of cognitive decline and AD is available (28–32).

Here, our aim was to investigate whether plasma NfL, GFAP, and pTau181 levels are associated with NPS and related long-term clinical outcomes, including the presence and the severity of NPS, and cognitive decline at follow-up visits. Furthermore, we evaluated the usefulness of the single plasma biomarkers or combinations of them to predict future NPS and cognitive decline. Additionally, we assessed whether the presence of NPS combined with measures of plasma NfL, GFAP and pTau181 improves the prediction of cerebral AD pathology as indicated by CSF biomarkers.

## METHODS

### STUDY POPULATION

We included 151 subjects from a brain aging study performed at the memory centers of the Department of Psychiatry and the Department of Clinical Neurosciences, University Hospital of Lausanne in Switzerland (33). All participants were community-dwelling older people with and without cognitive impairment recruited among memory clinic patients or through journal announcements and word of mouth. Cognitively impaired participants met the diagnostic criteria for MCI (34) and had a Clinical Dementia Rating (CDR) score of 0.5. Cognitively unimpaired individuals had a CDR score of 0. Exclusion criteria for all participants were current major psychiatric or neurological disorders, severe or instable physical diseases or substance use disorder, unstable medication and all medical conditions that may significantly contribute to cognitive impairment or interfere with cognitive performance in the administered tasks.

The study was conducted in accordance with applicable laws and regulations, including the International Conference on Harmonization, Guideline for Good Clinical Practice (35), and the ethical principles from the Declaration of Helsinki (36). The study was approved by the local ethics committee of canton Vaud, Switzerland (No. 171/2013). Prior to inclusion, written informed consent was obtained from all participants.

### CLINICAL AND NEUROPSYCHOLOGICAL ASSESSMENT

Comprehensive clinical and neuropsychological assessments were performed at baseline and at follow-up visits with the participants and their proxies. Cognitive performance was assessed using a comprehensive neuropsychological test battery as previously described (33). This battery included the domains memory, language, attention, as well as, executive and visuospatial functions. The instrumental activities of daily living (IADL) questionnaire assessed the functional impairment of each participant (37, 38). The clinical assessment, including physical, neurological, and psychogeriatric examination, and all described neuropsychological assessments were used to determine the CDR and CDR sum of boxes (CDR-SB) scores (39, 40). Cognitive impairment was defined as CDR = 0 and cognitive decline as CDR-SB > 0.5 (41), whereas the baseline CDR-SB score was subtracted from the CDR-SB score at follow-up. All tests and scales are validated and widely used in the field.

NPS at baseline and the follow-up visits were assessed using the Neuropsychiatric Inventory-Questionnaire (NPI-Q) (10). This is a self-administered questionnaire completed by informants with regular contact with the individual. It includes twelve domains (delusions, hallucinations, agitation/aggression, dysphoria, anxiety, euphoria, apathy, disinhibition, irritability/lability, aberrant motor activity, nighttime behavioral disturbances, and appetite/eating changes). Each domain was scored separately based on its severity, rated with values from one to three. The sum of the twelve scores yielded the total NPI-Q severity score, whereas the maximum score is 36. The presence of NPS was defined as NPI-Q > 0 at baseline or follow-up (i.e., future NPS). Based on the presence of any NPS at baseline, participants were divided into two subgroups: NPS positive (NPI-Q > 0) and NPS negative (NPI-Q = 0). The change in the NPS severity was investigated using the ΔNPI-Q severity score between baseline and follow-up, whereas the baseline NPI-Q score was subtracted from the NPI-Q score at follow-up.

### BIOLOGICAL ASSESSMENTS

Venous and lumbar punctures were performed between 8:30 and 9:30 am after overnight fasting at the recruiting centers. Ten to twelve milliliters of CSF were collected for analysis in a polypropylene tube, using a standardized technique with a 22G atraumatic spinal needle. Routine cell count and protein quantification were performed. CSF and plasma samples were centrifuged at 4°C, immediately aliquoted, and frozen at − 80°C until assayed.

Plasma NfL concentrations were measured using the NF-light^™^ kit on a Single molecule array (Simoa) HD-X Analyzer (Quanterix, Billerica, MA, USA), following the recommendations by the manufacturer. Plasma pTau181 levels were measured using an inhouse Simoa assay (21). Briefly, an AT270 mouse monoclonal antibody (MN1050; Invitrogen, Waltham, MA, USA) was coupled to paramagnetic beads (103,207; Quanterix) and used for capture. As the detector, we used the anti-tau mouse monoclonal antibody Tau12 (806,502; BioLegend, San Diego, CA, USA), conjugated to biotin (A3959; Thermo Fisher Scientific, Waltham, MA, USA), while GSK-3β phosphorylated full-length recombinant tau441 (TO8–50FN; SignalChem, Vancouver, BC, Canada) was used as calibrator. Fluorescent signals were converted to average enzyme per bead numbers, and specimen concentrations extrapolated from four-parametric logistic curves generated with known calibrator concentrations. Plasma GFAP was measured using a second generation simple plex GFAP assay (ProteinSimple, CA, USA) on an ultrasensitive microfluidic platform (Ella, Bio-Techne, Minneapolis, USA), according to the manufacturers’ instructions (42). Intra-assay coefficients of variation were less than 10%. Inter-assay imprecision was evaluated through repeating the measurement in six random samples with a mean variance of 11.2% (lowest 2.2% and highest 19.3%).

CSF Aβ_42_, total tau (tTau), and pTau181 concentrations were measured using commercially available ELISA kits (Fujirebio Europe, Gent, Belgium). The presence of CSF AD pathology was defined as a pTau181/Aβ_42_ ratio > 0.078, reflecting the concomitant presence of amyloid and tau pathology. This center cut-off was previously defined using study site data and is in line with previous publications (43). It was further confirmed by using longitudinal clinical follow-up data and comparing it to the literature as previously described (44).

For the genotyping of the apolipoprotein E (APOE) ε2/ε3/ε4 polymorphism, DNA was extracted from whole blood using the QIA symphony DSP DNA Kit (Qiagen, Hombrechtikon, Switzerland). Single nucleotide variation rs429358 and rs7412 were genotyped using the TaqMan assays C___3084793_20 and C____904973_10, respectively (Thermo Fischer Scientific, Waltham, MA). Participants with one or more ε4 allele were classified as carriers.

### DATA PREPARATION

Outliers (mean ± 3 standard deviations) were adjusted to the value of the nearest non-outlier (n = 2 for each of the three biomarkers). For missing value imputation, we used the mean of the non-missing values for replacement (n = 0 for NfL n = 2 for pTau181, and n = 23 for GFAP).

### STATISTICAL ANALYSIS

Descriptive statistics and regression analysis were performed using SPSS (IBM Corporation, Version 29.0, Armonk, NY, USA). For categorical variables, we calculated absolute and relative frequencies. For continuous variables, we calculated means and standard deviations. For cohort characteristics, two-tailed t-test and Mann-Whitney-U-test were applied for continuous and Pearson’s Chi-squared test for categorical variables. Normality of each variable was tested using the Shapiro-Wilk test to determine the appropriate statistical test. All statistical models were verified for possible overfitting using the Hosmer-Lemeshow test for goodness-of-fit. Models with a Hosmer-Lemeshow chi-squared value yielding a p-value > 0.05 were rejected and the previous iteration was considered instead. The alpha value was set at 0.05 for all statistical tests.

### ASSOCIATIONS OF PLASMA BIOMARKERS WITH CURRENT NPS, FUTURE NPS, AND NPS SEVERITY CHANGE

We used a binary definition for the presence of NPS (NPI-Q > 0) at baseline and at follow up (i.e., future NPS) to assess the relationship between the plasma biomarkers and the presence of NPS at baseline and follow-up. Furthermore, we used the NPI-Q severity score at baseline and at follow-up as well as their difference to assess associations of the plasma biomarkers with NPS severity change over time. The presence of NPS at baseline and future NPS were used as dependent variables within binary logistic regression analysis, while NPS severity at baseline and at follow-up as well as NPS severity change were used within linear regression analysis. Plasma NfL, GFAP, and pTau181 were set as independent variables. Age and sex were added to the models as covariates. Furthermore, the same approach was used stratifying based on cognitive status (cognitively impaired with CDR = 0.5 vs. cognitively unimpaired with CDR = 0 at baseline). In a further exploratory step, we also added AD status as a variable to determine possible confounding effects.

### ASSOCIATIONS OF PLASMA BIOMARKERS WITH COGNITIVE AND FUNCTIONAL DECLINE AND AD CEREBRAL PATHOLOGY

The binary definitions of cognitive and functional decline (CDR-SB > 0.5) and cerebral AD pathology were used as the dependent variables within binary logistic regression analysis. NfL, GFAP, and pTau181 were set as independent variables, while age and sex were added as covariates.

### PREDICTION OF FUTURE NPS

To assess the predictive performance of single markers, a combination of them and considering the presence of baseline NPS, we computed the area under the curve (AUC) of receiver operating characteristics (ROC) curves using the pROC package in R (45). Predictive models of future NPS (defined as NPI-Q > 0 at follow-up visit, indicating the presence of any NPS at follow-up (1, 46)) were built.

A convenience reference model including easily available clinical data, such as sex, age and baseline cognitive status, was built. The presence of baseline NPS and plasma NfL, GFAP and pTau181 - first all of them separately, then a combination of them - were added to the reference model. All models were then compared using the DeLong method (47). Additionally, sensitivity and specificity along with accuracy were calculated for the different models.

### PREDICTION OF COGNITIVE AND FUNCTIONAL DECLINE AD STATUS WITH PLASMA BIOMARKERS

To evaluate the performance of baseline NPS, single biomarkers, and combinations thereof to predict cognitive and functional decline or the presence of cerebral AD pathology, the same approach using ROC analysis as mentioned above was used.

## RESULTS

### STUDY PARTICIPANTS

Of the included 151 participants, 76 had normal cognition (CDR = 0) and 75 had mild cognitive impairment (CDR = 0.5). Clinical, demographic, and biological characteristics of the participants grouped by the presence/absence of NPS are shown in Table 1.

### ASSOCIATIONS OF PLASMA BIOMARKERS WITH CURRENT NPS, FUTURE NPS, AND NPS SEVERITY CHANGE OVER TIME

Results of the associations between plasma biomarkers and NPS at baseline, with future NPS and with NPS severity change are shown in Table 2.

### ASSOCIATIONS OF PLASMA BIOMARKERS WITH COGNITIVE AND FUNCTIONAL DECLINE AND AD PATHOLOGY

Higher levels of NfL were associated with cognitive and functional decline (OR 1.83, 95% CI 1.24–2.70, p = .002) and with the presence of cerebral AD pathology (OR 1.80, 95% CI 1.24–2.61, p = .002), but both associations lost significance after controlling for age. Higher levels of GFAP were associated with cognitive and functional decline (OR 1.56, 95% CI 1.07–2.27, p = .022), but the association lost significance after controlling for age. The association of higher GFAP with AD pathology remained significant after controlling for age and sex (OR 1.26, 95% CI 1.12–1.41, p < .001).

While pTau181 showed no association with cognitive and functional decline, higher levels of pTau181 were associated with AD pathology after controlling for age and sex (OR 1.07, 95% CI 1.03–1.12, p = .002).

### PREDICTION OF FUTURE NPS

**Figure 2:**
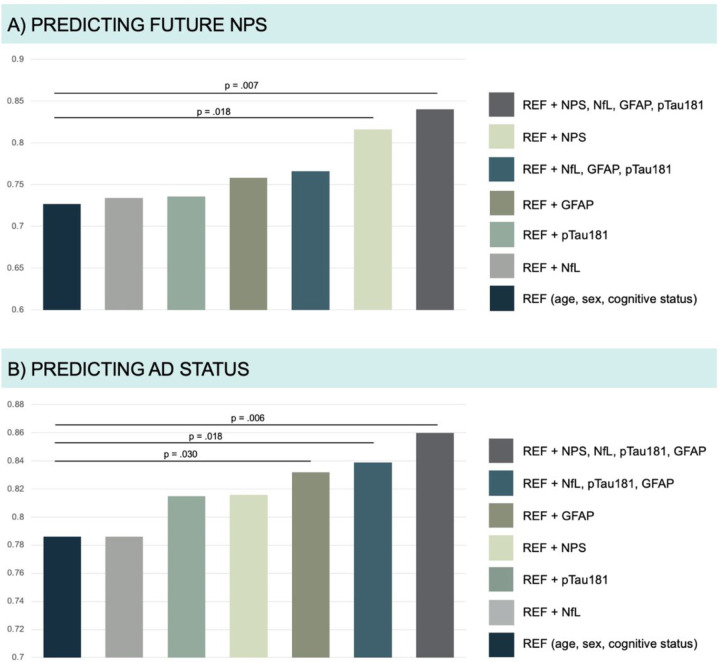
Predictive models for future NPS (A) and AD status (B) Results from the ROC-analysis, whereas each bar corresponds to the AUC value (y-axis) of a prediction model. Different single markers and combinations of them, compared with a reference model based on clinical data only, are shown. The legend on the right shows’ which markers are included in each model. Future NPS was defined as NPI-Q severity score > 0, cognitive status as either cognitive impairment (CDR=0.5) or cognitively unimpaired (CDR=0) and AD status as center cut-off pTau181/Aβ_42_ ratio < 0.078. The P-values above indicate significantly improved models. Aβ_42_, beta-amyloid 1–42 peptide; GFAP, glial fibrillary acid protein; NfL, neurofilament light chain; NPI-Q, neuropsychiatric inventory questionnaire; NPS, neuropsychiatric symptoms; pTau181, tau phosphorylated at threonine 181

To evaluate the utility of the plasma biomarkers alone, in combination, and considering the presence of baseline NPS we performed ROC analysis. When added to the reference model, the combination of baseline NPS and the three plasma biomarkers together improved the prediction of future NPS (from AUC 0.73 to 0.84, p = .007), as shown in Fig. 2A).

For the prediction of future NPS, the reference model had a specificity of 63%, a sensitivity of 78% and an accuracy of 71%, while the best model including NPS and the three plasma biomarkers improved to 73%, 73% and 77%, respectively.

### PREDICTION OF COGNITIVE AND FUNCTIONAL DECLINE AND AD PATHOLOGY

No single marker or combination was able to predict cognitive and functional decline at follow-up (from AUC 0.79 (reference model) to 0.84 (model including age, sex, cognitive status, the presence of NPS at baseline and plasma NfL, GFAP, and pTau181), p = .068). However, the combination of the three plasma biomarkers with the presence of NPS at baseline was the best model to predict cerebral AD pathology and improved prediction when added to the reference model (from AUC 0.79 to 0.86, p = .006). While the reference model had a specificity of 74%, a sensitivity of 68% and an accuracy of 71%, the best model improved to 89%, 71% and 82%, respectively (see Fig. 2B).

## DISCUSSION

Out of the three plasma biomarkers, only GFAP was associated with the NPI-Q severity score at baseline. Both higher plasma NfL and GFAP levels were associated with NPS at follow-up and with an increase in NPS severity over time. Considering NPS along with the three plasma biomarkers improved the prediction of future NPS, but not with cognitive and functional decline, as compared to a reference model based on clinical features. In addition, considering the presence of NPS along with the plasma biomarkers improved the prediction of cerebral AD pathology as defined using CSF biomarkers.

Higher plasma GFAP was associated with NPS severity at baseline. Additionally, higher GFAP was associated with the presence of NPS at follow-up and with an increase of the NPI-Q severity score over time. Only few studies investigated the relation between GFAP and NPS in a COVID context so far (48), and only one study in the context of AD (28). In the latter study the association between glial markers (plasma GFAP and microglial activation measured by TSPO-PET) and the NPI-Q score were investigated in a longitudinal research cohort including individuals with normal cognition, MCI, AD dementia, or other types of dementia. While plasma GFAP showed no association with NPS in this study, microglial activation in different brain regions as measured by TSPO-PET was associated with NPS. GFAP is considered a marker of astrocytic cell activation which is related to neuroinflammation in AD (49). Also neuroinflammation has been associated with NPS before (28, 50, 51), further supporting the idea of GFAP as a potential biomarker for NPS, and NPS progression over time. Our results suggest that GFAP may be a useful marker to predict NPS evolution over time. Additional studies are needed to further address the relationships between neuroinflammation, increased GFAP levels, and NPS, especially in the context of neurocognitive disorders.

Plasma NfL was not associated with the presence of NPS at baseline in our cohort. This is in line with the few existing studies investigating associations of NPS with NfL concentrations in plasma or CSF (30, 52, 53). However, a recent study in a cohort of individuals at clinical stages from cognitively unimpaired to dementia reported associations of plasma NfL with some single symptoms, such as aberrant motor behavior, anxiety, sleep disturbance, disinhibition, and euphoria (29). Of note, only amyloid positive individuals were included, which could have biased the results. A possible explanation for the lack of a clear association of NfL with NPS is that axonal degeneration as indicated by increased NfL levels may be not specifically related to NPS and also occur in the absence of NPS. However, higher NfL was associated with the presence of NPS at follow-up and with increasing NPS severity scores at follow-up, indicating a possible value as prognostic marker. To our knowledge, previous studies have not investigated NfL in relation to the evolution of NPS over time. Higher plasma NfL at baseline may indicate higher intensity of neurodegenerative processes which may more likely lead to the development of NPS. Our results suggest that higher plasma NfL indicates a higher risk of having (more severe) NPS in the future.

Plasma pTau181 was not associated with baseline NPS. This finding is in line with a recent study in a similar cohort (29). In our study, plasma pTau181 was also not related to NPS at follow-up. In a previous study using data from the ADNI cohort NPS were only associated with plasma pTau181 in participants having NPS at two different visits within one year (defined as mild behavioral impairment (5)), but not in those having NPS at only one visit (31). A few studies addressed the relationship between CSF pTau181 and NPS. While in a longitudinal study in cognitively unimpared older people, CSF pTau181 was correlated with higher NPI-Q scores after one-year (54), other studies did not find an association (32, 55, 56). Comparing these findings is difficult due to the different methods and populations considered (e.g., memory-clinic setting vs. research cohorts). Overall, our results, along with the previous ones, suggest that plasma pTau181 is not closely related to NPS and it may not be an useful marker for NPS and NPS evolution over time.

We further showed that considering the presence of baseline NPS along with plasma NfL, GFAP, and pTau181 improved the prediction of future NPS at follow-up visit. The presence of NPS at baseline showed the strongest contribution to the prediction model. To our knowledge, no previous study has investigated the potential of plasma biomarkers combined with NPS to predict the evolution of NPS over time.

Our findings suggest that plasma NfL, GFAP, and pTau181, together with easily accessible clinical assessments such as the NPI-Q may be helpful to predict future manifestation of NPS.

No single marker or combination with NPS was able to predict cognitive and functional decline. This may be related to the strong reference model in our study including age and baseline cognitive impairment, which both are known to be related to more rapid cognitive decline.

The combination of plasma NfL, GFAP, and pTau181 with NPS was the best model to predict cerebral AD pathology. These results show the importance of capturing NPS in memory-clinic settings to improve diagnosis and inform decision-making on further diagnostics and treatment. If confirmed, NPS along with cognitive measurements and easily accessible blood biomarkers could be of particular importance for decision-making on additional diagnostic steps.

There are some limitations to our study. We considered the presence of overall NPS, without differentiating between single symptoms. However, considering single symptoms would result in too small subgroups in our sample. Furthermore, we have not included participants with dementia or more severe NPS that may strongly interfere with cognitive performance. Accordingly, our findings may not be fully representative for the older people presenting with NPS. Plasma NfL, GFAP, and pTau181 were solely measured at baseline, wherefore no longitudinal changes in their levels were available. However, our study is to our best knowledge the first one investigating the associations between plasma NfL, GFAP, and pTau181 levels and the longitudinal evolution of NPS in older people with and without MCI. Including and following up participants that were cognitively unimpaired or at mild stages of cognitive impairment represents a strength of this study. This allows for assessing the potential contribution of NPS in relation to plasma biomarkers for early diagnosis and prognosis, which is of particular interest in the memory clinic patients.

## CONCLUSION

Our findings indicate that capturing NPS along with plasma NfL, GFAP, and pTau181 in memory clinic patients may be helpful to predict the evolution of NPS. Considering these findings could also help to improve personalized decision-making on further diagnostics and treatment while using non-invasive assessment methods. Further work is needed to confirm and extend these observations, considering specific NPS and additional blood-based biomarker candidates, to improve the prediction of the NPS manifestation and evolution over time.

## Figures and Tables

**Figure 1:**
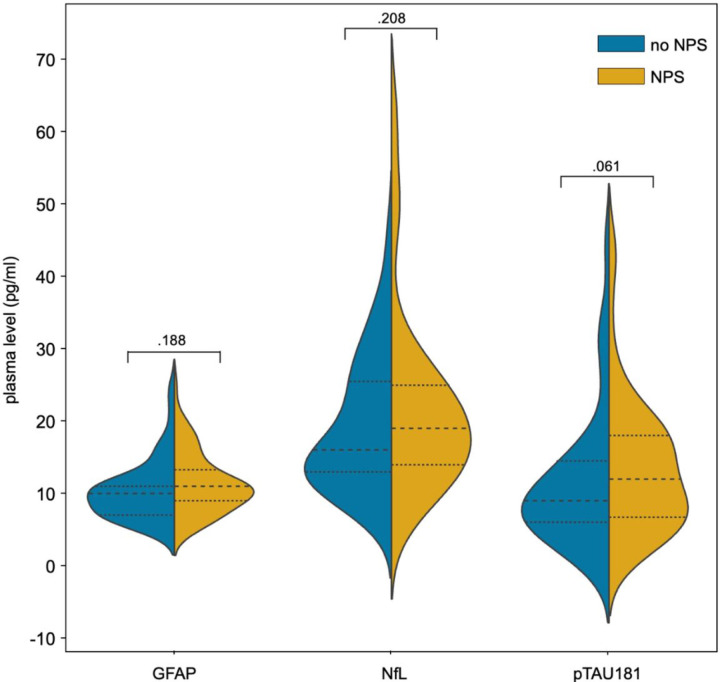
Plasma levels of GFAP, NfL and pTau181 at baseline in participants with and without NPS Violin plot showing the concentration (in pg/mL, y-axis) of plasma GFAP, NfL and pTau181 (x-axis) grouped by the presence (orange) or absence (blue) of NPS defined as NPI-Q >0. The dashed lines indicate the mean, and the dotted lines indicate the standard deviation. P-values from group comparison are shown above each plot. GFAP, glial fibrillary acid protein; NfL, neurofilament light chain; NPI-Q, neuropsychiatric inventory questionnaire; NPS, neuropsychiatric symptoms; pTau181, tau phosphorylated at threonine 181

**Table 1:** Characteristics of the study participants grouped by the presence of NPS at baseline Criteria for the NPS positive group (NPS +) were NPI-Q score > 0, for the NPS negative group (NPS -) NPI-Q score = 0. Mean values ± standard deviation are shown. A positive AD profile was defined based on a center cut-off of pTau181/Aβ_42_ ratio < 0.078. Cognitive impairment was defined as CDR = 0.5. Aβ_42_, beta-amyloid 1–42 peptide; APOEe4, Apolipoprotein E epsilon 4; AD, Alzheimer’s disease; CDR, clinical dementia rating; CDR-SB, clinical dementia rating sum-of-boxes; GFAP, glial fibrillary acid protein; MMSE, mini mental status examination; n, number; NfL, neurofilament light chain; NPI-Q, neuropsychiatric inventory questionnaire; NPS, neuropsychiatric symptoms; pTau181, tau phosphorylated at threonine 181 Participants with NPS were older and had more marked impairment in global cognition (i.e., lower mini mental status examination (MMSE) scores) and higher disease severity (i.e., higher CDR and CDR-SB scores). In addition, the presence of cerebral AD pathology as indicated by a CSF AD biomarker profile was more frequent in participants with NPS. The distributions of the CSF core AD biomarkers between the two groups are shown in supplementary **Table S1**. The plasma biomarker levels did not differ between the participants with and those without NPS at baseline (see Fig. 1). The mean NPI-Q severity score at baseline was 5.0 ± 4.6. In those having NPS the most common symptoms were anxiety (51.4%), apathy (41.7%), sleeping disorders (38.9%), irritability (36.1%), depression (31.9%) and eating disorders (31.9%) (for all frequencies see supplementary **Table S2**). Follow-up data on NPS was available in 108 participants and on cognitive impairment in 137 participants.

	total n = 151	NPS + n = 72	NPS – n = 79	p
**Demographic data**				
sex, female (%)	85 (56.3)	35 (48.6)	50 (63.3)	.074
age, years	71.0 ±7.4	72.3 ±6.3	69.8 ±8.1	.034
education, years	12.8 ±2.7	12.5 ± 3.0	13.0 ± 2.5	.315
**Clinical data**				
cognitive status, impaired (%)	77 (55.4)	49 (76.6)	26 (32.9)	< .001
CDR	0.2 ±0.3	0.3 ±0.2	0.2 ±0.2	< .001
CDR-SB	0.7 ±1.0	1.2 ±1.2	0.3 ±0.6	< .001
MMSE	27.3 ±2.9	27.9 ±2.8	26.5 ± 3.0	< .001
CSF AD profile (%)	59 (39.3)	42 (59.2)	17 (21.5)	< .001
APOEe4 carrier (%)	55 (34.7)	31 (45.6)	19 (25.0)	.014
plasma pTau181 (pg/ml)	12.5 ± 9.3	13.5 ± 9.2	11.5 ± 9.4	.061
plasma NfL (pg/ml)	20.5 ± 10.9	21.9 ± 12.4	19.2 ± 9.2	.208
plasma GFAP (pg/ml)	10.8 ± 4.1	11.2 ± 4.1	10.4 ± 4.1	.188
follow-up time (months)	19.8 ± 15.1	19.9 ± 10.7	19.7 ± 18.2	.155

**Table 2:** Associations of plasma biomarkers with NPS severity and NPS severity change Results from the linear regression analysis showing the associations of plasma NfL, GFAP and pTau181 with NPS severity at baseline and follow-up (based on the NPI-Q total severity score) as well as the NPS severity change over time (defined through the ΔNPI-Q total severity score between baseline and follow-up) after considering age and sex. Beta coefficients, 95% confidence interval and p-values are shown. GFAP, glial fibrillary acid protein; NfL, neurofilament light chain; NPI-Q, neuropsychiatric inventory questionnaire; NPS, neuropsychiatric symptoms; pTau181, tau phosphorylated at threonine 181 Only GFAP was associated with the NPI-Q severity score at baseline. This association was no longer significant after considering AD pathology (**Table S3**). Higher NfL and higher GFAP levels were associated with future NPS at follow-up and with an increase of the NPI-Q severity score (see **Figure S1**) over time. These associations remained significant after considering AD pathology (**Table S3**). Results stratified based on cognitive impairment are shown in **Table S4**.

	baseline NPS severity		future NPS severity		NPS severity change	
	β (95% CI)	p	β (95% CI)	p	β (95% CI)	p
NfL	0.06 (−0.13–0.24)	.543	0.29 (0.08–0.51)	.007*	0.23 (0.02–0.45)	.035*
GFAP	0.23 (0.06–0.40)	.008*	0.28 (0.08–0.48)	.007*	0.27 (0.06–0.47)	.011*
pTau181	0.09 (−0.07–0.26)	.272	0.18 (−0.01–0.37)	.068	0.09 (−0.11–0.29)	.365

## Data Availability

The datasets used and/or analyzed during the current study are available from the corresponding author on reasonable request.

## Abbreviations

Aβ42: beta-amyloid 1–42 peptide
AD: Alzheimer’s disease
ADNI: Alzheimer’s Disease Neuroimaging Initiative
APOEe4: Apolipoprotein E epsilon 4
AUC: area under the curve
CDR: clinical dementia rating
CDR-SB: clinical dementia rating sum-of-boxes
CI: con dence interval
CSF: cerebrospinal fluid
GFAP: glial fibrillary acid protein
IADL: instrumental activities of daily living
MCI: mild cognitive impairment
MMSE: mini mental status examination
NfL: neurofilamet light chain
NPI-Q: Neuropsychiatric Inventory Questionnaire
NPS: neuropsychiatric symptoms
pTau181: tau phosphorylated at threonine 181
ROC: receiver operating characteristics
Simoa: single molecule array
tTau: total tau
